# Feeder delivery vehicle scheduling optimization of high-speed railway express based on trunk and branch intermodal transportation

**DOI:** 10.1038/s41598-022-16560-1

**Published:** 2022-07-27

**Authors:** Yao Cui, Xiaoye Zhou

**Affiliations:** 1 School of Management, Shenyang University of Technology, Shenyang, 110000 China; 2 School of Management, Liaoning Institute of Science and Technology, Benxi, 117000 China

**Keywords:** Sustainability, Applied mathematics, Computer science

## Abstract

In view of the traditional branch line end express delivery centralized mode cannot adapt to the growing demand of high-speed rail (HSR) express, resulting in poor connection between trunk and branch line, high cost and poor timeliness. In this paper, the problem of scheduling optimization of branch-line flexible distribution vehicles relying on intermodal transportation of trunk and branch lines is proposed. Considering the number of vehicles, vehicle capacity, customer service time window and other constraints, an integer linear programming mathematical model with the minimum total cost of vehicle transportation cost, usage cost and time window penalty cost as the optimization objective is established. A two-level nested heuristic algorithm with two-level coding structure is proposed to solve the problem. Finally, a simulation example is given to verify the effectiveness of the model and the algorithm. The results show that the vehicle scheduling optimization problem studied in this paper can effectively improve the timeliness and accuracy of HSR express delivery, and can significantly reduce the total vehicle delivery cost.

## Introduction

The High-Speed Rail (abbreviated hereinafter as HSR) express service is proposed to fulfill the consumer's requirements for timeliness and accuracy of express delivery^1^, As the HSR is fast, punctual, safe, and not affected by traffic congestion and environmental changes, it can provide small volume, multi-frequency, high timeliness, and high value-added services for fragmented cargo.

HSR completes “station to station” express delivery through railway trunk network, and the vehicles at the end of branch lines provide “station to door” service through the distribution network of express companies, to realize the combined transportation and distribution of HSR and vehicles. Among them, the transfer connection is very important to the whole combined transportation and distribution process. When the time and space of the connection are mismatched, the vehicles transportation resources are seriously wasted, and the time limitation of express delivery is affected. Therefore, it is an urgent problem for express intermodal transport enterprises to solve how to effectively connect terminal delivery vehicles with HSR.

By 2021, more than 200 cities in China have opened HSR express services such as same-city express, cross-city express and same-day express. The passenger and freight synergy mode are used in HSR to transport urgent goods, high added value, cold chain, fresh and other goods at existing dispatching scheme and idle transport capacity.

By 2021, more than 200 cities in China have opened HSR express services, such as same-city express, cross-city express and same-day express. HSR adopts a collaborative passenger and cargo model, using existing scheduling solutions and idle capacity to transport urgent cargo, high value-added, cold chain, fresh and others.

At present the distribution mode of HSR express transport still adopts the traditional centralized distribution mode, i.e., HSR express transport cargo is stored centrally in the warehouse of HSR station, and then regularly centralized distribution by truck. However, this model takes up storage space, cannot respond to customer demand in time, and wastes the speed and time advantage of HSR, resulting in its same effect as traditional railroad transportation without improving timeliness, although it increases the cost of HSR transportation. The other is the customized distribution mode for special cargo, i.e., dispatching trucks immediately after the arrival of HSR express cargo. This mode can respond quickly to fulfill the demand of high timeliness, but the increase cost of the vehicle is not the result we want.

Therefore, the design of a flexible HSR express delivery mode has become inevitable, according to the different arrival times of HSR express, the cargo is decomposed or integrated in a ladder according to the time window demand. Based on existing research and the reality of HSR express, we look for a better intermodal distribution mode suitable for modern HSR express, design mathematical models and algorithms, and solve and verify them with simulation examples.

Therefore, for the sake of improving the efficiency and reducing the cost of HSR express, a feeder distribution model relying on trunk-branch intermodal transportation and an optimal scheduling strategy for feeder distribution vehicles are proposed in this paper based on the actual situation. This mode is a Vehicle Routing Problem with Soft Time Window considering Trunk-branch Transfer Connection Dispatching (VRPSTWTTCD). The essential difference from traditional VRPSTW is that it needs to consider the departure time, scale, and route of the delivery vehicle by the batch arrival time and quantity of HSR express.

There are few researches on VRPSTW problem of HSR express. Pazour^2^ considered the factors of HSR freight transport capacity and road transport time, and constructed the design model of highway-railway intermodal transport network. Ertem et al.^3^ put forward the fast transportation by HSR network for small-sized goods and mail, and set up the high-speed trains scheduling optimization model. Wang Baohua et al.^4^ studied the location and Transportation Service Network optimization of express freight transportation network based on HSR and highway. Zufferey et al.^5^ set up the transport planning and route selection model of the rail-road intermodal transport, and the route follows the truck-rail-truck intermodal transport model, and uses Tabu search algorithm to solve the model. Santos et al.^6^ proposed an innovative mixed-integer programming model based on hub-and-spoke theory to improve the competitiveness of rail-rail combined transport by optimizing the location of freight terminals. Walha et al.^7^ constructed the optimization model of the distance between truck and freight station and the number of trucks used, and proposed a multi-agent based on heuristic. Saeed et al.^8^ studied the distribution center layout and branch line distribution path optimization in multi-modal transport network are studied, and an improved genetic algorithm is designed to solve them. Xie Haihong et al.^9^ put forward the distribution model of the Railway Logistics Distribution Center of the highway-railway intermodal transportation, and established the route choice model with the lowest logistics cost of the customer as the goal.

In the research of traditional VRP, some scholars have studied the integration of vehicle routing problem and vehicle dispatching problem. Ozbaygin et al.^10^ studied the impact of customer demand dynamics on vehicle routing and departure schedule, and designed branch and bound algorithm to solve the problem. Zhang Dezhi et al.^11^ constructed the joint optimization model of vehicle departure time and vehicle routing problem, and used two-stage hybrid genetic algorithm to solve the two sub-problems of vehicle departure time and vehicle routing problem. Zhou Lin^12^ studied the integrated optimization problem of vehicle routing and dispatching, and solved it with a hybrid evolutionary search algorithm combining genetic algorithm and local search. Liu, H. et al.^13^ presented an integrated decision-making of multi-vehicle type combined strategy and route optimization based on customer demand, and an improved genetic algorithm is designed.

## Model building

### Problem description

The number of HSR is $R$, arriving at the station $D$. The number of distribution vehicles assigned to each train trip varies greatly due to the influence of HSR passenger traffic. The amount of express delivery delivered varies greatly with the remaining capacity of each HSR. If customized vehicle is assigned to pick up and transport each arrival express, because of the capacity of the vehicle cannot be matched optimally, it will result in a high rate of vehicle empty operation, a serious waste of vehicle resources and an increase in distribution costs. If centralized distribution is adopted for the arrival express, as the batch of express arrived has to wait for centralized and unified distribution, the waiting time is long, and it cannot be delivered in time as required by the time limit.

Therefore, it is necessary to determine the number of vehicles for each express delivery on HSR from the perspective of vehicle loading rate for all-day express delivery, and to distribute the selected express delivery according to the demand of the customer time window. The feeder delivery vehicles depart from the HSR station at the same time. When visiting the customer point, the service time is allowed to be advanced and delayed, but corresponding punishment must be given. The vehicles return to the station after distribution. For the remaining express delivery, unified planning shall be made with the next batch.

The goal of the optimization is how to determine the dispatching plan of the delivery vehicles according to the loaded of each train. Considering factors such as the number, capacity of freight vehicles, and the service time window of customer, a mathematical model is established to minimize the total cost included by transportation, Vehicle use cost and penalty cost of the time window. In order to determine the distribution, the scale, the departure time, and the running route of express delivery vehicles, and realize the effective connection between terminal delivery vehicles and HSR.

### Model assumption

1. HSR operation plan, remaining capacity, location coordinates of HSR stations and customers, customer demand and time window are known.
2. All HSR trains can carry freight, and the freight loaded on each train does not exceed the minimum residual capacity of the HSR.
3. Transfer time of HSR express is fixed.
4. The HSR station has sufficient temporary storage service capacity.
5. The vehicle models are the same and the carrying capacity is certain.
6. Each vehicle can satisfy the needs of multiple customers, and each customer can only be served by one vehicle.
7. The time window penalty cost standard for each customer is the same.

### Symbolic description

The notations and meanings used in model construction are shown in the Table 1.

1 $$\min L = C_{1} \sum\limits_{i,j \in P} {\sum\limits_{v \in K} {\sum\limits_{r \in R} {d_{ij} x_{ijv}^{{t_{r} }} y_{v}^{{t_{r} }} } } } + C_{2} \sum\limits_{r \in R} {z_{r} } + \sum\limits_{i \in P} {\sum\limits_{v \in K} {\sum\limits_{r \in R} {\left[ {A\max (E_{i} - h_{i} ,0) + B\max (h_{i} - F_{i} ,0)} \right]} } y_{v}^{{t_{r} }} }$$

**Table 1:** Definitions of notations.

Name	Description
$I$	Set of arrival time of HSR
$t_{r}$	Departure time of vehicles $t_{r} \in I$
$R$	Set of trains $r \in R$
$G$	Set of rail station $O,D \in G$
$P$	Set of customers $i,j,l \in P$
$K$	Set of vehicles
$q_{i}$	Demand of customer $i$
$d_{ij}$	Distance from node $i$ to $j$
$\left[ {E_{i} ,F_{i} } \right]$	Service time window, earliest starting time and latest starting time
$S_{i}$	Service time for node $i$
$h_{i}$	Starting service time to node $i$
$t_{ij}$	Travel time from node $i$ to $j$
$t_{Di}$	Travel time from Station ${\text{D}}$ to node $i$
$W_{v}$	Capacity of the vehicle
$C_{1}$	Transportation cost per kilometer of vehicle
$C_{2}$	Use cost of the vehicle
${\text{A}}$	Waiting cost per hour of vehicle
${\text{B}}$	Penalty cost per hour of vehicle
$C_{\max }$	Maximum penalty cost
${\text{N}}_{\max }$	Maximum number of vehicles
${\text{T}}$	Transit time of the vehicle
${\text{M}}$	A large positive integer
$u_{iv}^{{t_{r} }}$	0–1 decision variable representing whether the client $i$ is traveled by vehicle $v$ departed from time $t_{r}$
${\text{z}}_{r}$	Decision variable representing the number of vehicles
$y_{rv}^{{t_{r} }}$	0–1 variable representing whether the train $r$ is transferred by vehicle $v$ departed from time $t_{r}$
$x_{ijv}^{{t_{r} }}$	0–1 variable representing whether the path $(i,j)$ is traversed by vehicle $v$ departed from time $t_{r}$

s.t.

2 $$\sum\limits_{\begin{subarray}{l} i \in P \\ i \ne j \end{subarray} } {\sum\limits_{v \in K} {x_{ijv}^{{t_{r} }} y_{v}^{{t_{r} }} = 1} } ,\quad \forall r \in R,\;j \in P,\;t_{r} \in I$$

3 $$\sum\limits_{i \in P} {\sum\limits_{v \in K} {u_{iv}^{{t_{r} }} q_{i} } } \le W_{v} \sum\limits_{v \in K} {z_{r} y_{v}^{{t_{r} }} } ,\quad \forall r \in R,\;t_{r} \in I$$

4 $$\sum\limits_{\begin{subarray}{l} l \in P \\ l \ne i \end{subarray} } {x_{ljv}^{{t_{r} }} } = \sum\limits_{\begin{subarray}{l} i \in R \\ i \ne j \end{subarray} } {x_{ijv}^{{t_{r} }} } ,\quad \forall r \in R,\;j \in P,\;v \in K,\;t_{r} \in I$$

5 $$h_{i} = \varepsilon_{r} + T + t_{Di} + \left( {1 - x_{ijv}^{{t_{r} }} } \right)M,\quad \forall i,j \in P,\;r \in R,\;v \in K,\;t_{r} \in I$$

6 $$h_{j} = \left( {h_{i} + S_{i} + t_{ij} } \right)x_{ijv}^{{t_{r} }} ,\quad \forall i,j \in P,\;r \in R,\;v \in K,\;t_{r} \in I$$

7 $$\varepsilon_{r} + T + \left( {1 - u_{iv}^{{t_{r} }} } \right)M \le t_{r} y_{v}^{{t_{r} }} ,\quad \forall i \in P,\;r \in R,\;v \in K,\;t_{r} \in I$$

8 $$\sum\limits_{r \in R} {z_{r} } \le N_{\max }$$

9 $$\sum\limits_{i \in P} {\sum\limits_{v \in K} {\sum\limits_{r \in R} {\left[ {A\max (E_{i} - h_{i} ,0) + B\max (h_{i} - F_{i} ,0)} \right]} } } \le C_{\max }$$

10 $$u_{iv}^{{t_{r} }} \in \left\{ {0,1} \right\},\quad \forall i \in P,\;r \in R,\;v \in K,\;t_{r} \in I$$

11 $$x_{ijv}^{{t_{r} }} \in \left\{ {0,1} \right\},\quad \forall i,j \in P,\;r \in R,\;v \in K,\;t_{r} \in I$$

12 $$y_{v}^{{t_{r} }} \in \left\{ {0,1} \right\},\quad \forall r \in R,\;v \in K,\;t_{r} \in I$$

13 $$z_{r} \ge 0,\quad \forall r \in R$$

The objective function (1) represents the lowest total cost of transportation and distribution. Constraint (2) guarantees that the feeder delivery vehicle visits each customer only once, and the customer demand cannot be divided. Constraint (3) prevent the express delivery volume exceed the total loading capacity of the feeder delivery vehicle. The flow conservation is defined in (4) indicates that the number of vehicles entering a customer point is equal to leaving. Formulas (5) and (6) are defined as the arrival time or service start time from node $i$ to node $j$. Constraint (7) ensures that the actual departure time of vehicles is later than the arrival time of HSR. Constraints (8) and (9) represent the limit of the maximum vehicle that can be used and the limit of the maximum penalty cost. Constraints (10)–(12) are the integer constraint of the model decision variable 0–1. Constraint (13) indicates non-negative constraint on the number of vehicles.

## Two-level nested ant colony optimization algorithm with two-level coding structure

Ant Colony Optimization (abbreviated hereinafter as ACO) algorithm has been widely used to solve combinatorial optimization problems with good robustness and positive feedback^14–16^.

In view of the specific points of the problem studied in this paper, based on ACO algorithm, a two-level nested ant colony search strategy was designed by nesting and reverse recursion, and the two-level coding structure was improved to solve the problem.

Express of each HSR are allocated to vehicles according to the principle of large journey interception algorithm which sorted customers according to the time window, and allocated vehicles according to capacity limit. The express exceeding the capacity limit are allocated together with the express of the next batch as the same principle^17^. In order to avoid local convergence of the algorithm, a mutation disturbance mechanism is designed, that two customers are randomly exchanged from the current solution, and re-optimized with the constraint of capacity, and set the disturbance frequency as 1/3 of the number of iterations to improve the operation efficiency.

**Figure 1:**
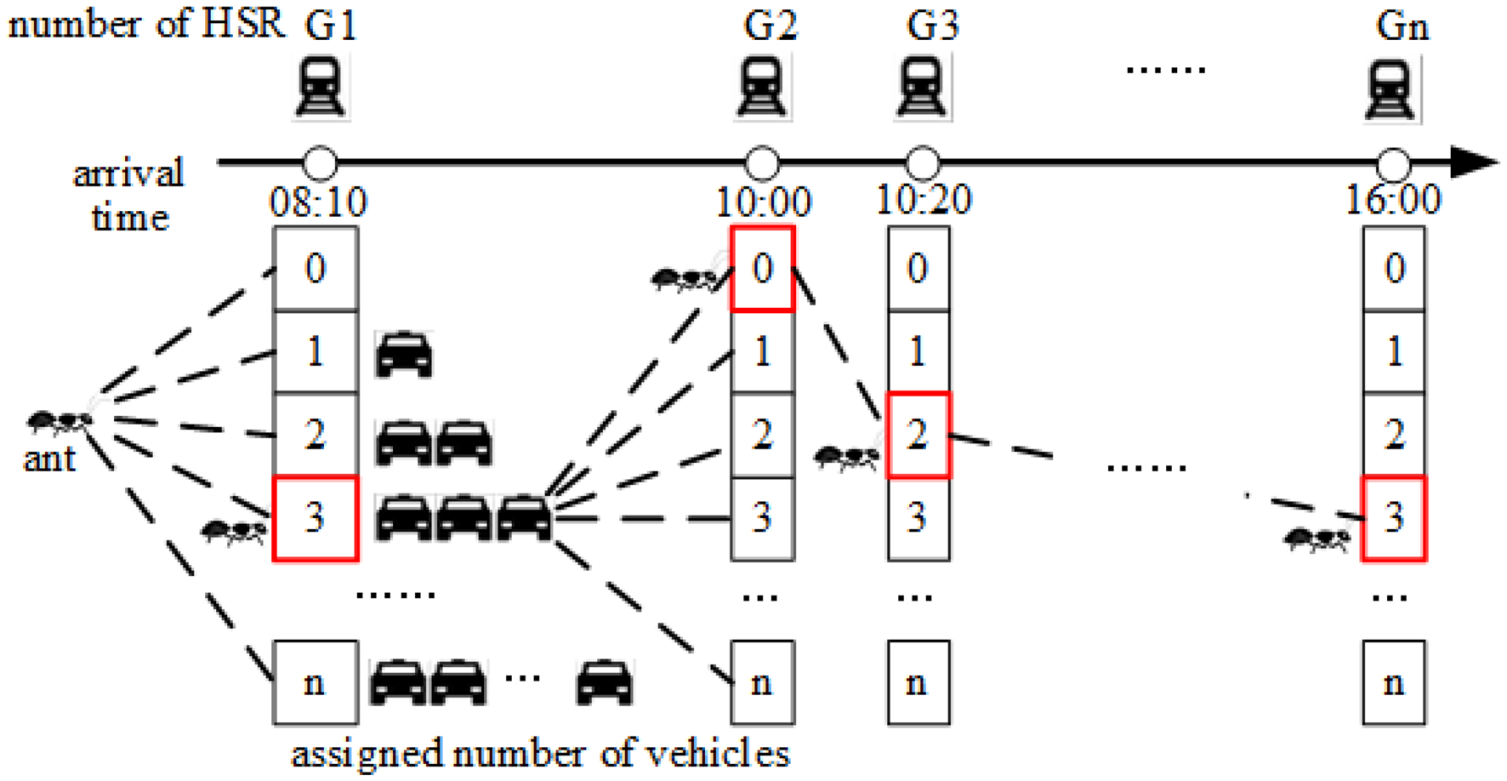
Schematic diagram of node selection in the first layer of improved ACO algorithm.

Figure 1 shows that the ant crawling principle of the first level improved ACO algorithm. The number of vehicles instead of the location is used as the path node visited by the ant. The ant selects the next visited node according to the pheromone concentration, and considers the vehicle loading rate in the pheromone heuristic factor.

### Coding structure

Firstly, the assigned number of vehicles is considered as the path node visited by the ants, replacing the original location node^18^. And the nodes are visited according to the arrival times of high-speed trains.

**Figure 2:**
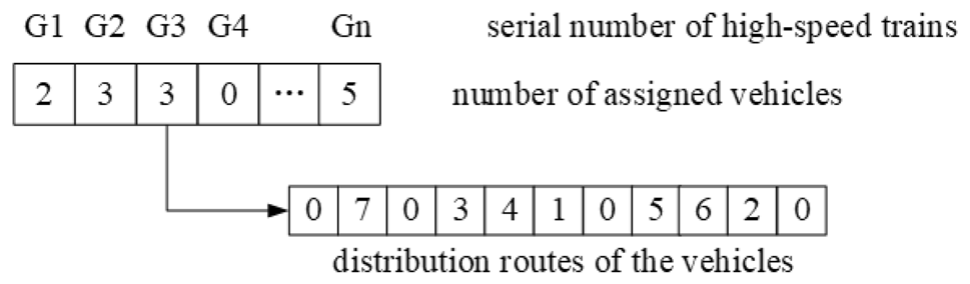
Schematic diagram of two-level coding structure.

Secondly, the digits in upper-tier are defined as the number of assigned vehicles per HSR, 0 represents no vehicle is assigned for the current HSR, and in lower-tier are defined as customer serial number and the route of each vehicle. The coding structure is as shown in Fig. 2. The number of vehicles allocated to HSR numbered (G1, G2, G3, G4…, Gn) are (2, 3, 3, 0…, 5) respectively, and the distribution routes of vehicles of G3 are 0 → 7 → 0; 0 → 3 → 4 → 1 → 0; 0 → 5 → 6 → 2 → 0.

### Selection probability

The path $(i,j)$ represents the number of scheduled vehicles is $j - 1$ for the train $i$; and $p_{ij}^{k}$ represents the probability of ant $k$ select path $(i,j)$.

14 $$p_{ij}^{k} = \left\{ {\begin{array}{*{20}l} {\frac{{\tau (i,j)^{\alpha } \eta (i,j)^{\beta } }}{{\sum\limits_{{j \in \left[ {{1,}v_{i} + 1} \right]}} {\tau (i,j)^{\alpha } \eta (i,j)^{\beta } } }},} \hfill & {j \in \left[ {{1,}v_{i} + 1} \right]} \hfill \\ {0,} \hfill & {others} \hfill \\ \end{array} } \right.$$

**Figure 3:**
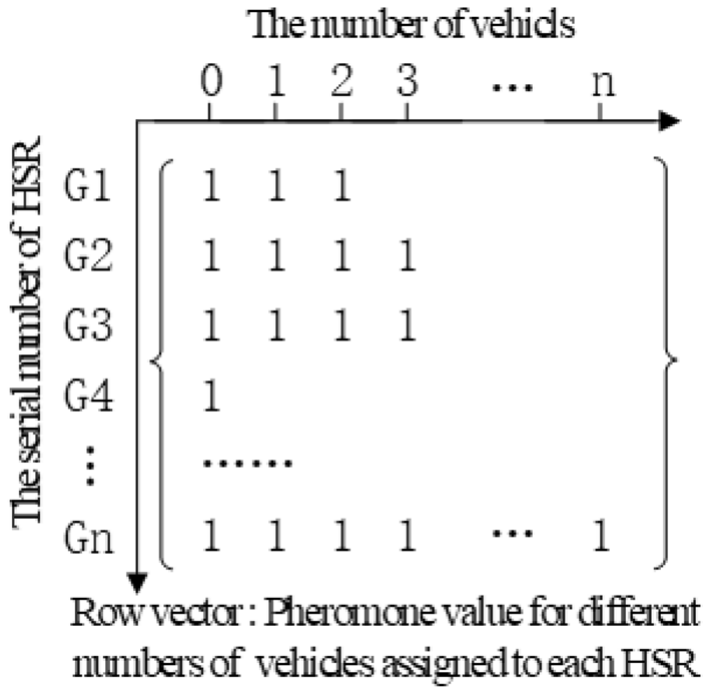
Pheromone concentration tuple structure.

Two-dimensional unequal length array is generated by using tuple as the pheromone concentration, rows represent the number of delivery vehicles, and the columns is the series number of each HSR. The initial value of pheromone concentration set 1 as the Fig. 3 shows.

15 $$\tau_{t} (i,j) = (1 - \rho )\tau_{t - 1} (i,j) + \sum\limits_{k = 1}^{m} {\Delta \tau_{t - 1}^{k} (i,j)}$$

Formula (15) is defined as the update of node pheromone concentration, $\Delta \tau_{t - 1}^{k} (i,j)$ represent pheromone increment left by the ant $k$ on path $(i,j)$ in iteration $t - 1$,and $\Delta \tau_{t - 1}^{k} (i,j) = \frac{Q}{{V_{k} }}$, $Q$ is enhancement coefficient, $V_{k}$ means the total cost of the ant $k$ after accessing all nodes.

The heuristic factor plays an important role in ant searching to better nodes, which effectively promotes the convergence of the algorithm. As showed in formula (16), the vehicle loading rate is used as the heuristic factor to replace the original distance.

16 $$\eta \left( {i,j} \right) = \left\{ {\begin{array}{*{20}l} {\frac{{Q_{i} }}{{W_{j} }},} \hfill & {when\;0 < \frac{{Q_{i} }}{W} < 1,} \hfill \\ {\frac{{W_{{\left( {j - 1} \right)}} }}{{Q_{i} }},} \hfill & {when\;j < \left\lceil {\frac{{Q_{i} }}{W}} \right\rceil + 1,} \hfill \\ {\frac{{Q_{i} }}{{W_{{\left( {j - 1} \right)}} }},} \hfill & {when\;j \ge \left\lceil {\frac{{Q_{i} }}{W}} \right\rceil + 1.} \hfill \\ \end{array} } \right.$$

### Improvement on tabu table of ACO algorithm

Traditional ACO algorithm records the visited nodes according to tabu table, which prevents the repeated selection from getting stuck in an endless cycle. This section designs double tabu table structure: the global tabu table and the reverse recursive local tabu table. Pseudocode 1 demonstrates the process of tabu table. The global tabu table is used to record the infeasible solution which not satisfies the constraint conditions, and to prevent the algorithm from getting stuck in the endless cycle and local optimization. The reverse recursive local tabu table is used to correct the repeated unfeasible solutions in this iteration. When the route is in the global tabu table, do the correction operation by the recursive procedure.
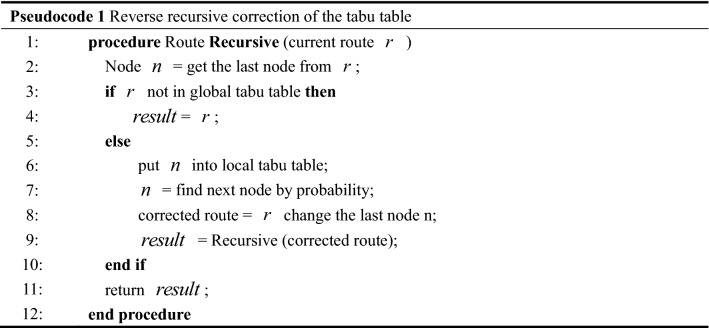


### Algorithm description

Pseudocode 2 details the steps involved in the two-level nesting algorithm. Step1, initializes global tabu table, population size, iteration times and sets the pheromone concentration tuple. Setp2, calculates the node selection probability of the ant $m$ by formula (14), and initializes the local tabu table. Setp3, generates solution route by the selection probability. Step4, invokes the reverse recursive procedure to correct the solution shown in Pseudocode 1. Judging whether the current solution satisfies the constraint condition and outputs the solution.
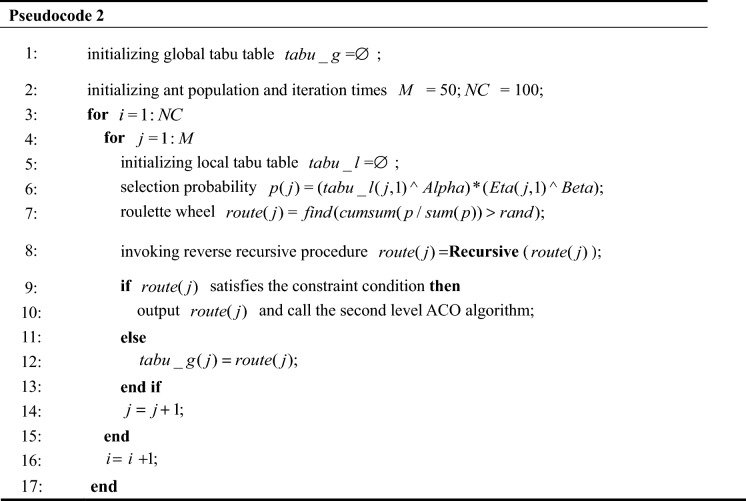


The complexity of the algorithm is affected by the iterations $NC$, population size $M$, client count $N$, and the number of high-speed trains $R$.

### Spatial complexity analysis

The memory space occupied by the upper-tie ACO algorithm is $M \times R$, and the lower is $\sum\limits_{i = 1}^{R} {M \times N_{i} }$, so the space complexity is $O\left( {R \times N \times M^{2} } \right)$.

### Time complexity analysis

The time complexity of the upper-tie ant colony algorithm includes the time complexity of the selection probability $O\left( N \right)$ and the time complexity of the traversal node $O\left( R \right)$, so the time complexity is $O\left( {N \times M \times NC} \right) + O\left( {R \times M \times NC} \right)$. And the lower-tie complexity includes the time complexity of choosing probability $O\left( N \right)$, the time complexity of traversing nodes $O\left( {\frac{{N\left( {N - 1} \right)}}{2}} \right)$, so the time complexity is $O\left( {\frac{{N\left( {N - 1} \right)}}{2} \times M \times NC} \right)$.

In summary, the total time complexity is

$$O\left( {\frac{{N\left( {N - 1} \right)}}{2} \times M \times NC} \right) \times \left[ {O\left( {N \times M \times NC} \right) + O\left( {R \times M \times NC} \right)} \right] \approx O\left( {\frac{{N\left( {N - 1} \right)}}{2} \times M^{2} \times NC^{2} \times R} \right)$$

The results show that the time complexity of the nested algorithm is $O\left( {n^{7} } \right)$. It is related to the number of customer nodes, and closely related to the number of trains of HSR. With the increase of the number of trains and the number of customer nodes, the problem scale increases to the 7th power.

## Simulation experimentation and analysis

In this section, we use three simulation examples to validate the performance of the modal and algorithm. The first one calculates the results of the model optimization in this paper and compares it with centralized delivery and customized delivery. The second tests the efficiency of the algorithm for different scales. At last, compares the results with tabu search and genetic algorithm. The steps of the simulation experiment are overviewed as follows.

1. *Data generation* Randomly generate simulation data with reference to the characteristics of the actual user requirements.
2. *Data parsing* Analyze and classify the simulation data.
3. *Parameter setting* Configure the experimental environment and the basic parameters of the ACO algorithm.
4. *Model calculation* Based on the same environment and data, the model designed in this paper and the traditional model are calculated and output.
5. *Result analysis* The experimental results are compared and analyzed, and conclusions are drawn.

### Simulation experiment

We go get a total of 8 high-speed trains from 7:00 to 14:00, 40 customer points, and randomly generate their time windows, demand, and geographical locations, all of which go through a uniform distribution. The HSR station is used as the center of the interchange with coordinates of (43.59, 82.85). The maximum residual capacity and the arriving time of each train are obtained founded on the analysis of historical passenger traffic data.

The maximum number of vehicles available for distribution is 30, the fixed use cost of vehicles is 30 per vehicle, the driving cost of vehicles is 2 per kilometer, and the average speed of vehicles is 15 km/h, early time window penalty costs 10 per hour, late time window penalty costs 20 per hour. Setting the distribution vehicle rated capacity is 1 ton by limiting traffic in the city.

As shown in Table 2, the source of the simulation experiment data and the description of the fields are as follows:

**Table 2:** Known simulation information of HSR and customers.

Train	Arrival time	Max capacity/t	Total demand /t	Client point	Location (X, Y)	Demand/t	Time window
G1	7:02	2.1	2.03	1	49.26, 72.04	0.36	8:00–9:00
				2	30.01, 89.19	0.06	9:00–10:00
				3	44.74, 87.58	0.21	9:30–10:30
				4	35.07, 71.34	0.1	7:30–8:30
				5	30.35, 90.88	0.75	9:30–10:30
				6	57.12, 80.27	0.05	8:30–9:30
				7	42.41, 90.01	0.09	7:30–8:30
				8	48.97, 79.60	0.06	7:30–8:30
				9	47.45, 80.68	0.3	7:30–8:30
				10	30.66, 87.11	0.05	8:30–9:30
G2	7:20	0.4	0.31	11	35.61, 73.22	0.31	8:00–9:00
G3	7:27	1.4	1.37	12	32.94, 74.50	0.68	8:30–9:30
				13	35.60, 79.25	0.47	8:30–9:30
				14	43.31, 88.80	0.21	8:00–9:00
				15	35.70, 74.49	0.01	9:00–10:00
G4	9:16	0.4	0.34	16	56.45, 78.16	0.24	11:30–12:30
				17	31.62, 82.30	0.1	11:30–12:30
G5	10:27	2.5	2.35	18	30.57, 89.16	0.18	12:00–13:00
				19	38.06, 77.04	0.56	11:00–12:00
				20	46.32, 77.84	0.18	11:00–12:00
				21	44.77, 89.05	0.07	11:00–12:00
				22	47.17, 84.94	0.9	11:00–12:00
				23	59.50, 91.94	0.46	12:00–13:00
G6	10:50	4.4	4.12	24	46.76, 86.55	0.18	11:00–12:00
				25	33.70, 73.56	0.91	11:30–12:30
				26	49.43, 77.16	0.74	11:30–12:30
				27	51.36, 80.73	0.6	11:30–12:30
				28	45.05, 84.62	0.3	11:00–12:00
				29	32.97, 82.70	0.89	11:30–12:30
				30	49.60, 77.80	0.2	11:30–12:30
				31	34.34, 76.87	0.3	15:00–16:00
G7	13:24	3.0	2.84	32	48.77, 90.78	0.52	14:00–15:00
				33	35.18, 81.72	0.91	14:00–15:00
				34	46.23, 71.22	0.13	14:00–15:00
				35	37.68, 71.84	0.44	14:30–15:30
				36	33.55, 92.18	0.33	14:30–15:30
				37	58.96, 88.28	0.51	15:30–16:30
G8	13:55	2.3	2.1	38	40.65, 91.85	0.34	14:30–15:30
				39	32.38, 85.89	1.0	14:30–15:30
				40	52.44, 72.84	0.76	14:30–15:30

Column 1, 2 indicates the number of HSR trains and the arriving time, which is obtained from the actual train schedule. Column 3 indicates the maximum cargo loading capacity that can be allowed for each high-speed train under the condition that passenger transportation is fulfilled. Column 4 indicates the total freight volume of the current HSR train, i.e., the total customer demand of the current train, which should be less than or equal to the maximum freight loading capacity, otherwise the exceeding part needs to be allocated to the next train for transportation. Columns 5–8 indicate the location coordinates, demand, and time window of customers respectively, which are randomly generated by the program and satisfy with uniform distribution.

Parameters of the Algorithm are set as follows: population size $M = 50$, maximum iteration times $NC_{\max } = 100$, pheromone concentration weights $\alpha = 1$, heuristic factor weight $\beta = 0.8$, pheromone concentration volatile coefficient $\rho = 0.75$, pheromone concentration enhancement coefficient $Q = 100$, programmed with MATLAB R2016a, and run on a microcomputer with quad-Core processor (2.0 GHZ) and 16 GB memory.

**Figure 4:**
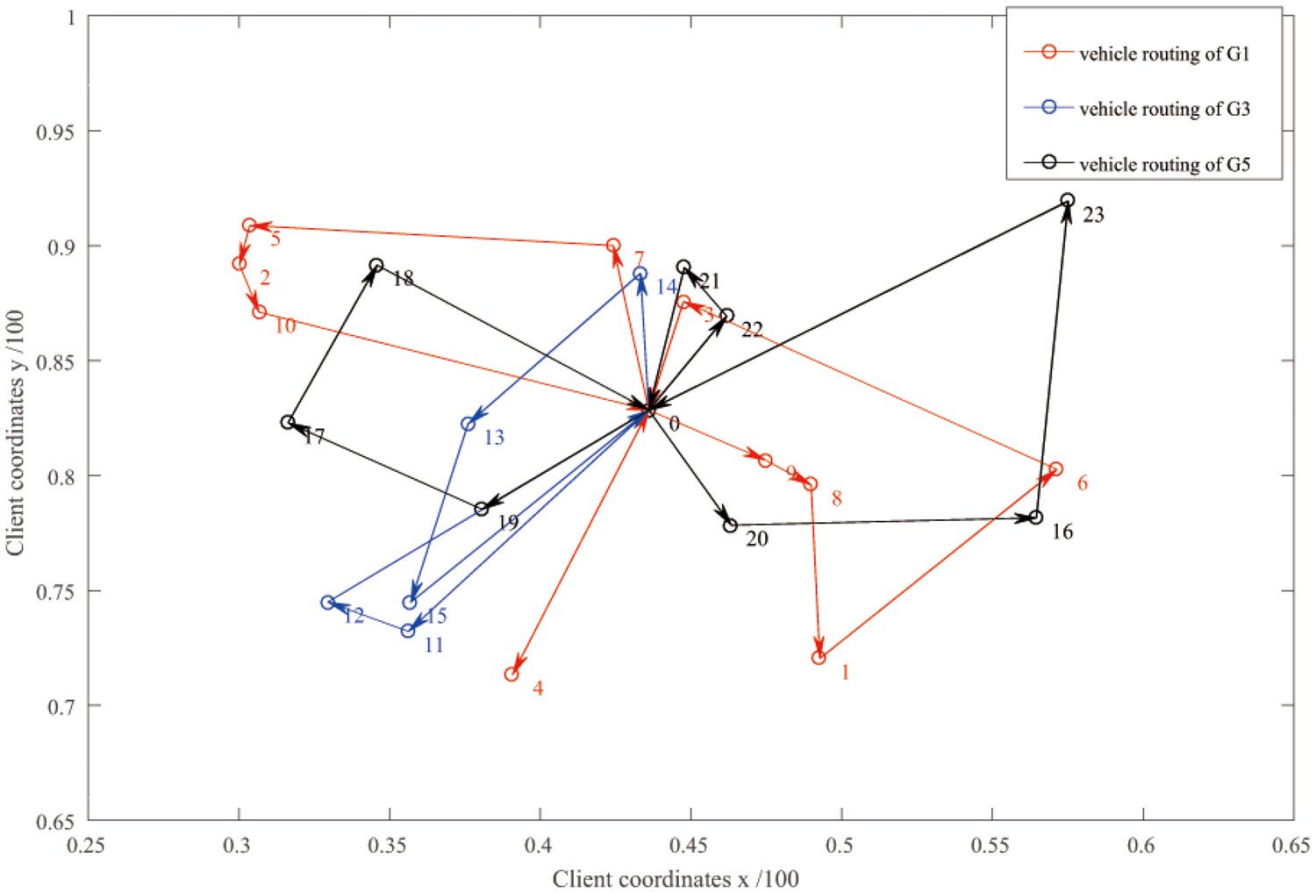
Distribution route optimization of vehicle for HSR G1, G3, G5.

**Figure 5:**
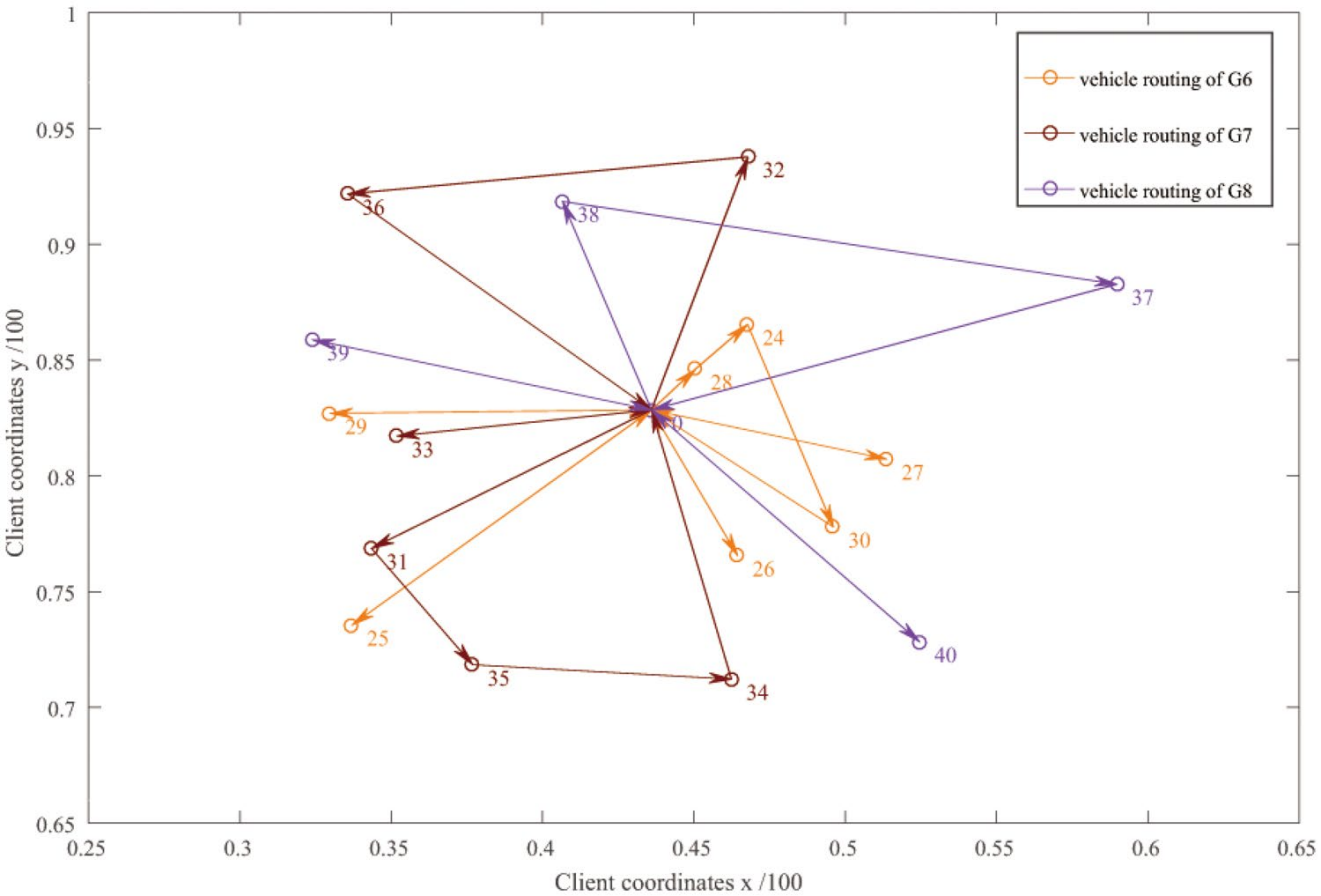
Distribution route optimization of vehicle for HSR G6, G7, G8.

By means of a perturbation mechanism and an improved algorithm, the optimal solution for express and vehicle scheduling of HSR is obtained. Figures 4 and 5 shows the operation results of vehicle routing optimization algorithm, and compared with the results of centralized delivery and customized delivery in Table 3.

**Table 3:** Running detail results of simulation experiment and comparison with centralized delivery and customized delivery.

Mode	Start time	Vehicle number	Path	Loading rate (%)	Outside time window	Total cost
Flexible delivery	7:12	3	0 → 9 → 8 → 1 → 6 → 3 → 0 0 → 7 → 5 → 2 → 10 → 0 0 → 4 → 0	81.05	1-early	1050.8921
	7:30	0				
	7:37	2	0 → 14 → 13 → 15 → 0 0 → 11 → 12 → 0			
	9:26	0				
	10:37	3	0 → 20 → 16 → 23 → 0 0 → 19 → 17 → 18 → 0 0 → 22 → 21 → 0			
	11:00	5	0 → 28 → 24 → 30 → 0 0 → 27 → 0, 0 → 26 → 0 0 → 29 → 0, 0 → 25 → 0			
	13:34	3	0 → 34 → 35 → 31 → 0 0 → 32 → 36 → 0 0 → 33 → 0			
	14:05	3	0 → 38 → 37 → 0 0 → 39 → 0, 0 → 40 → 0			
Centralized delivery		17		90.59	3-early 18-late	1878.1323
Customized delivery		27		57.04	8-early	1425.4848

From the experimental results, the departure time of vehicles in flexible distribution mode varies with the arrival of HSR expresses dynamically. Each row in Table 3 corresponds to one HSR trip. The starting time of the delivery vehicle of G1 HSR express is 7:12, the number of optimally dispatched vehicles is 3, and the vehicle paths are 0 → 9 → 8 → 1 → 6 → 3 → 0, 0 → 7 → 5 → 2 → 10 → 0, and 0 → 4 → 0. 0 indicates the distribution center (HSR station), and the digits are customer numbers.

Although G2 HSR express cargo exists for 11, the optimized number of vehicles is 0. This means G2's cargo will be integrated with the next train if the time window is fulfilled. Therefore, according to the situation of HSR express, the departure time and the number of flexible delivery vehicles can be obtained.

In the results of the experiment, for the vehicle loading rate, the centralized distribution model is the highest of 90.59%, the customized distribution model is the worst of 57.04%, and the flexible distribution model is in between of 81.05%.

In terms of the number of vehicles, the centralized delivery mode has the least number of vehicles of 17, the customized delivery mode has the most of 27, and the flexible delivery mode is in between of 19.

In terms of the number of deliveries outside the time window, the flexible delivery mode is the best at 1, earlier than the earliest time window, the centralized delivery mode is the worst at 21, with 3 early arrivals and 18 late arrivals, and the customized delivery mode, 8 early arrivals.

Therefore, the total delivery cost of the flexible delivery mode is 1050.8921, which is better than the other two modes. Mainly because the flexible distribution mode is processed by the integration of HSR express, which can generate the minimum penalty cost within the time window that is, as well as can improve the loading rate of the vehicle and reduce the cost of using the vehicle.

### Comparison with different scales

In this section, the problem scale is raised to 15 HSR and 60 clients, 20 HSR and 80 clients, 25 HSR and 100 clients respectively. The results calculated by the improved algorithm are given in Table 4. The performance and applicability of the improved algorithm can be analyzed from the optimal, inferior, and average values calculated by the algorithm, the running time of the algorithm, the searching success rate, and the average number of iterations.

**Table 4:** Comparison and analysis of running results of different scales.

Scale trains-customers	Simulation results					
	Best value	Worst value	Mean value	Run time	Success rate (%)	Average iterations
8–40	1050.8921	1485.2726	1195.3229	232.5147	39.5	16.43
15–60	1592.3795	1902.2751	1702.9482	249.4358	35.1	19.56
20–80	2151.6597	3514.1372	2576.6132	395.7993	29.6	28.25
25–100	3015.8478	5192.7466	4375.4614	917.7431	17.3	45.41

In Table 4, the first column is the scale of the experimental data (the number of high-speed trains and the number of customers), and when the problem size is 20–80, the difference between the optimal value of 2151.6597 and the worst value of 3514.1372 increases, and the average value of 2576.6132 deviates more in the direction of the worst value, and the success rate of the algorithm to find the optimal solution decreases to 29.6%, and the running time of the program is 395 s. The average number of iterations is 28.25. When the size of the problem continues to increase, the success rate of searching for the optimal solution decreases significantly, and the computation time and the number of iterations also increase significantly, mainly because of the time complexity of the nested algorithm for $O\left( {n^{7} } \right)$.

In summary, the improved algorithm is appropriate for solving the problem of scheduling HSR express pickup vehicles of medium and below scale, and has a strong global search capability. HSR express transportation is exactly the small and medium scale transportation, which is in line with the reality.

### Comparison with other algorithms

**Figure 6:**
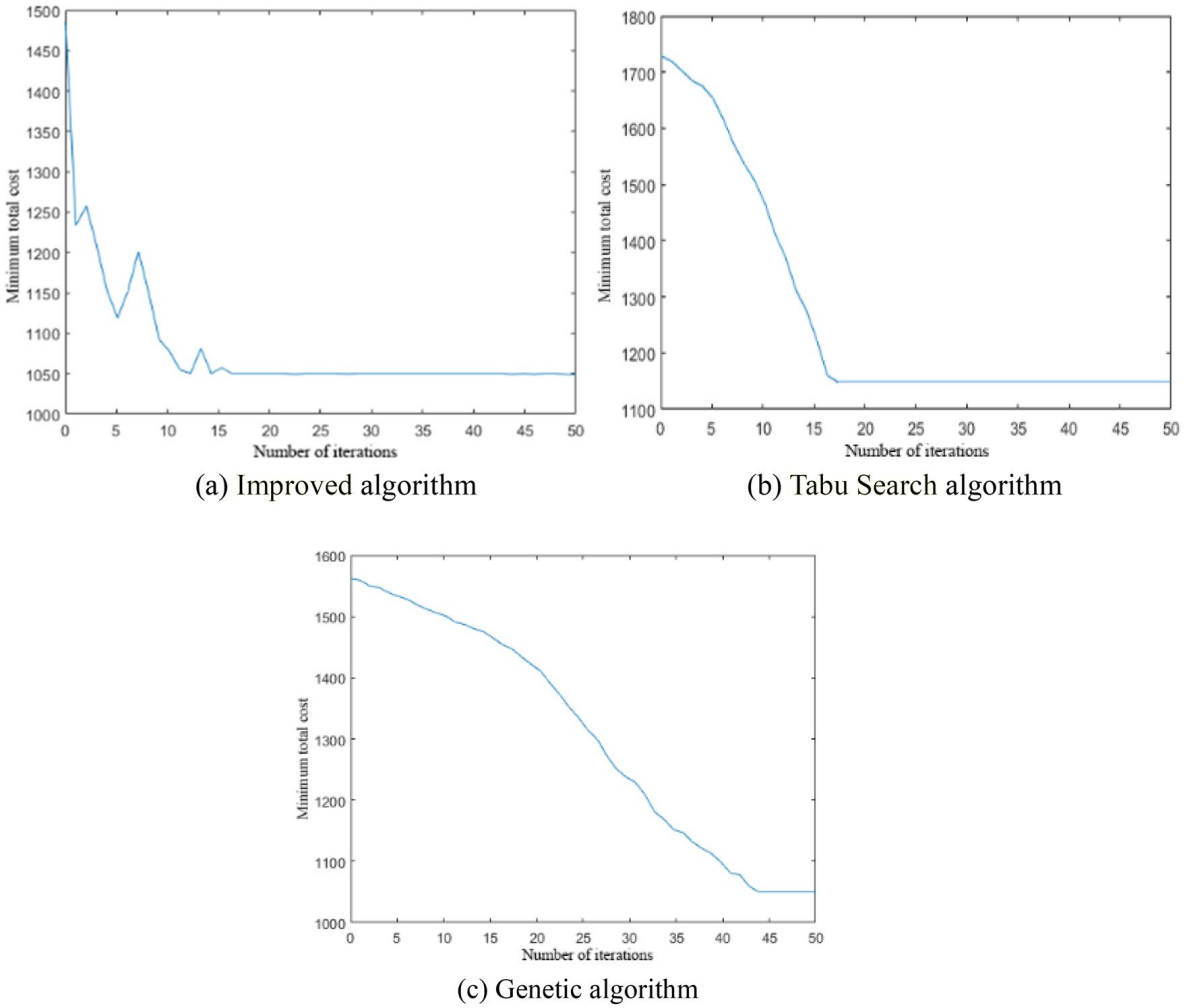
Convergence diagram of three algorithm.

In this section, we compare improved algorithm with advanced artificial intelligence algorithms, the Tabu Search algorithm (TS) and Genetic algorithm (GA). The results are computed with these three algorithms separately and recorded to compare and analyze the algorithms in terms of their search ability (optimal value, worst value, and average value), the running time of the algorithms, the search success rate, and the average number of iterations. Figure 6 shows the improved algorithm has better convergence, and the mean iteration times is 16.43.

From Table 5, the improved algorithm of this paper and Genetic algorithm can get the optimal solution, for 1050.8921, while the Tabu Search algorithm is poorer, for 1148.1342, and falls into a local optimum.

**Table 5:** Algorithm performance comparison analysis.

Algorithm	Best value	Worst value	Mean value	Run time	Success rate (%)	Mean iterations
Improved algorithm	1050.8921	1485.2726	1195.3229	232.5147	39.5	16.43
TS	1148.1342	1729.5436	1577.3823	180.9335	10.4	18.37
GA	1050.8921	1562.3413	1226.6167	358.2718	20.5	43.72

The worst value of the improved algorithm in this paper is smaller than the other two algorithms, and the average value is closer to the optimal value, indicates that the improved algorithm in this paper has better convergence characteristics.

Tabu search algorithm run time is the shortest, using only 180 s, the average number of iterations is also the smallest, for 18.37, but the success rate of the search is low, only 10.4%, and local convergence, cannot meet the expectations.

Through comparative analysis, the improve algorithm of this paper converges quickly, with rapid running time and high searching success rate, and can obtain a better optimal solution, which proves that the algorithm has obvious advantages over the other two heuristic algorithms in terms of convergence, applicability, and implementation efficiency. Reasons for this are that the improved algorithm in this paper adopts the global taboo table and reverse recursive taboo table search strategies to preprocess the upper ACO algorithm to minimize the search space while covering the feasible solutions. Thus, the efficiency and accuracy of the whole algorithm are improved.

## Conclusion

In this paper, the optimization problem of vehicle scheduling for feeder delivery of HSR express is studied, and a vehicle scheduling optimization model is established. Taking the number of vehicles instead of the location as the path node visited by ants, and the percentage of the remaining capacity of vehicles is added to the heuristic factor instead of distance. The optimal distribution scheme of HSR express and the vehicle scheduling scheme of feeder distribution are obtained.

The simulation results show that, compared with the traditional two modes of customized vehicle distribution and centralized distribution, the vehicle scheduling optimization problem studied in this paper can provide accurate distribution services, and greatly reduce the total cost of HSR express transportation and distribution. The improved algorithm designed in this paper can obtain a better solution and achieve rapid convergence.

However, the remaining capacity of HSR is greatly affected by passenger flow, and the information of HSR express is generated in real time. Therefore, considering the influence of dynamic HSR express loading scheme on intermodal vehicle scheduling will be the next research direction.

## Data Availability

All data generated or analyzed during this study are included in this article.
